# Adaptive Walking in Alzheimer's Disease

**DOI:** 10.1155/2012/674589

**Published:** 2012-09-09

**Authors:** Diego Orcioli-Silva, Lucas Simieli, Fabio Augusto Barbieri, Florindo Stella, Lilian Teresa Bucken Gobbi

**Affiliations:** ^1^Laboratório de Estudos da Postura e da Locomoção, Departamento de Educação Física, Universidade Estadual Paulista (UNESP), Avenida 24-A, 1515 Bela Vista, 13506-900 Rio Claro, SP, Brazil; ^2^UNICAMP, State University of Campinas, Av. Albert Einstein, 763 Cidade Universitária 13083-852 Campinas, SP, Brazil

## Abstract

The aim of this study is to analyze dual-task effects on free and adaptive gait in Alzheimer's disease (AD) patients. Nineteen elders with AD participated in the study. A veteran neuropsychiatrist established the degree of AD in the sample. To determine dual-task effects on free and adaptive gait, patients performed five trials for each experimental condition: free and adaptive gait with and without a dual-task (regressive countdown). Spatial and temporal parameters were collected through an optoelectronic tridimensional system. The central stride was analyzed in free gait, and the steps immediately before (approaching phase) and during the obstacle crossing were analyzed in adaptive gait. Results indicated that AD patients walked more slowly during adaptive gait and free gait, using conservative strategies when confronted either with an obstacle or a secondary task. Furthermore, patients sought for stability to perform the tasks, particularly for adaptive gait with dual task, who used anticipatory and online adjustments to perform the task. Therefore, the increase of task complexity enhances cognitive load and risk of falls for AD patients.

## 1. Introduction

 Elderly people with Alzheimer's disease (AD) show reduced gait performance [1], such as a slow and irregular stride [2]. Gait adjustments of patients with AD have been explained by frontal lobe dysfunctions, especially in the motor cortex [2, 3], and by an intense decrease in executive functions [3]. Moreover, patients with AD have a less automated gait [1, 4], especially when a concurring executive task (dual task) is performed. Furthermore, elderly people with AD are more prone to falls when compared to healthy elders [5], falling 4 to 5 times a year [6]. Studies have shown that touched or stumbled on the obstacles are one of the major causes for falls in AD patients [7]. 

 The studies of the dual-task effects during gait in AD patients have focused on free gait [8]. However, AD patients do not walk only on even terrain during their daily activities. They need to adapt their locomotor behavior to different travel surfaces. Dual-task effect on adaptive gait (walking characterized by presence of obstacles that demand adaptive strategies) in AD patients is poorly understood [9]. Adaptive gait competes with the executive task for attention and planning functions, especially due to previously observed relationships between performing complex motor tasks and executive functions [3, 10, 11]. Therefore, dual task during adaptive gait seems to be more challenging for AD patients.

 The aim of this study is to analyze dual-task effect on spatial and temporal parameters of free and adaptive gait in AD patients. We expect patients with AD to perform more adjustments during adaptive gait, adopting conservative strategies for crossing obstacles, such as bigger stride width and slower stride velocity than free gait and during gait with dual task, such as reduced balance and stride length. Still, accentuation of modulations is expected in adaptive gait with dual task.

## 2. Materials and Methods

### 2.1. Subjects

Thirty elderly people, who were diagnosed with AD according to the Diagnostic and Statistical Manual of Mental Disorders (DSM-IV-TR) [12], were volunteers in this study. Inclusion criteria, according to the clinical evaluation of dementia [13], were (i) patients had to walk independently and (ii) patients had to be classified either on the mild (1.0) or moderate (2.0) stages. Eleven elders with a severe impairment of cognitive functions did not fit the inclusion criteria. Therefore, the study sample was composed of 19 elders with AD (Table 1), divided into 14 women and five men.

### 2.2. Procedures

 This study was approved by the local ethics committee (no. 0739/2011). Experimental procedures were performed during two days. In the first day was performed a full anamnesis of the diseases, lesions and in-use medication to verify inclusion criteria. To characterize the AD degree of the patients, a veteran neuropsychiatrist (FS) applied the following evaluations: Clinical Dementia Rating scale [13]; Neuropsychiatric Inventory [14]; Mini-Mental State Examination [15]; Clock-Drawing Test [16]; the Frontal Assessment Battery [17].

 In the second day were performed the experimental tasks. AD patients performed 5 trials on the following tasks: free gait without dual task, free gait with dual task, adaptive gait without dual task, and adaptive gait with dual task. The secondary task performed by patients during gait was countdown from 20 to 1. Trials order was randomized for each patient. The instruction given to the patients was to walk over an 8 m pathway at self-selected velocity. For the adaptive gait trials, the participant was instructed to avoid contact with the obstacle (the height was half the leg size of each patient). For dual-task trial, the patients were also instructed to perform the countdown naturally and loudly.

 Acquisition of kinematic gait parameters was accomplished with a three-dimensional optoelectronic system (OPTOTRAK Certus 3D Motion Measurement System, NDI), positioned in the sagittal right plane, using a sample rate of 100 samples/s. Four infrared emitters were placed on the subjects' following anatomical landmarks: lateral face of calcaneus and head of 5th metatarsus of the right limb and the medial face of calcaneus and head of 1st metatarsal of the left limb.

### 2.3. Data Analysis

 Spatial and temporal parameters were calculated on MATLAB (The Math Works, Natick, MA, USA). Tridimensional data were filtered with a fifth-order Butterworth low-pass filter with cutoff frequency of 6 Hz. For free gait, we analyzed the stride in the middle of the pathway, which was compared to the stride preceding the obstacle crossing for adaptive gait (approach phase). For adaptive gait, we additionally analyzed the crossing step. During free gait and the approaching phase on adaptive gait, the stride length, stride duration, single and double support duration, step width, and stride velocity were measured. Particularly for the crossing stride on adaptive gait, we calculated the stride duration, stride velocity, single and double support duration, the foot-obstacle distance before and after obstacle crossing, and toe clearance for the leading and trailing limbs. Furthermore, we quantified countdown mistakes in executive task trials (forgetting or repeating numbers) and also obstacle contact events during adaptive gait. Trials with countdown mistakes were included in the analysis, while patients redid trials with any obstacle contact events.

 Spatial and temporal parameters were statistically analyzed on SPSS 15.0 for Windows. The dependent parameters of free gait and the approaching phase on adaptive gait in the experimental conditions were compared by ANOVA tests (*P* < 0.05) with repeated measures for experimental condition (free and adaptive gait with and without dual task). When univariate analyses revealed a main effect, Tukey post hoc tests were used to point out differences among experimental conditions (*P*-adjusted < 0.008). Similarly a paired Student's *t*-test (*P* < 0.05) was performed for spatial and temporal parameters of crossing step on adaptive gait with and without dual task. Countdown mistakes and obstacle contact events were expressed as percentage according to experimental conditions.

## 3. Results

 Amongst the 19 patients, sixteen elders showed a mild AD, while three (3) of them presented a moderate AD (Table 1). One subject was not able to perform dual task during gait, and another one was not able to perform the adaptive gait. They were not included in the analysis. Overall, patients performed 85 trials for each experimental condition. In free gait with dual task, patients performed countdown mistakes in 37.6% of the trials, while in adaptive gait with dual task patients performed mistakes in 44.7% of the trials. Obstacle contact events happened in 5.9% of dual-task trials. There were no obstacle contact events in adaptive gait without dual task.

**Figure 1:**
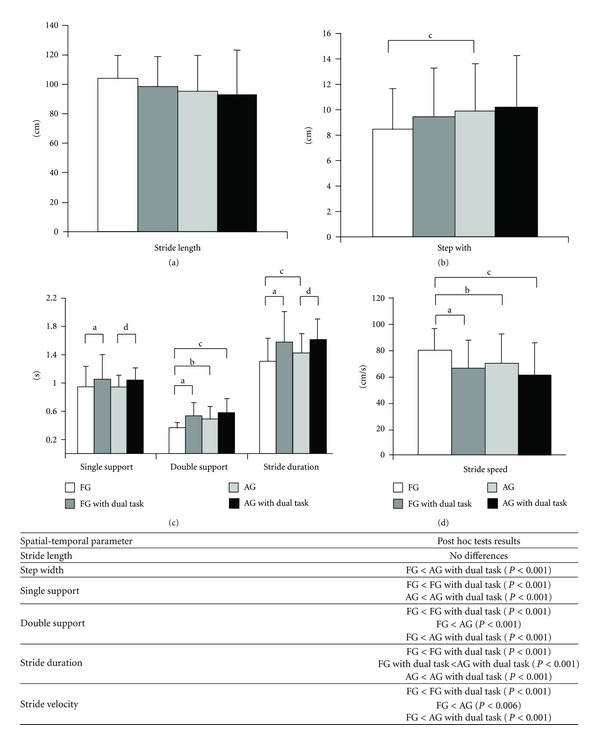
Mean and standard deviation of the spatial-temporal parameters for free and adaptive gait (approach phase). The table shows a summary of the significant difference for the parameters. FG: free gait without dual task; AG: adaptive gait without dual task. In the figure, (a) differences between free gait without dual task and free gait with dual task; (b) differences between free gait without dual task and adaptive gait without dual task; (c) differences between free gait without dual task and adaptive gait with dual task; (d) differences between adaptive gait without dual task and adaptive gait with dual task.

In free gait and approaching phase on adaptive gait, ANOVA revealed significant differences among conditions for step width (*F*_3,249_ = 4.49; *P* < 0.005), single support duration (*F*_3,249_ = 6.74; *P* < 0.001), double support duration (*F*_3,249_ = 27.62; *P* < 0.001), stride duration (*F*_3,249_ = 21.43; *P* < 0.001), and stride velocity (*F*_3,249_ = 15.16; *P* < 0.001). A summary of the post hoc test was showed in the table above Figure 1.

Regarding crossing stride parameters (Table 2), the paired *t*-test revealed a shorter stride duration (*t*_83_ = −5.25; *P* < 0.001), single support phase (*t*_83_ = −2.98; *P* < 0.004) and double support phase (*T*_83_ = −5.62; *P* < 0.001), as well as a higher stride velocity (*t*_83_ = 3.67; *P* < 0.001) during adaptive gait without dual task when compared to adaptive gait with dual task. Moreover, patients showed a higher toe clearance for the support limb (*t*_83_ = 2.21; *P* < 0.03) during adaptive gait without dual task.

## 4. Discussion

 The aim of this study was to analyze the dual-task effect on spatial and temporal parameters of free and adaptive gait in AD patients. The expectations of the study were confirmed, especially for adaptive gait with dual task. Patients used conservative strategies when confronted either with an obstacle or a secondary task decreasing gait velocity. Furthermore, patients sought for stability to perform their tasks, particularly the most complex one (adaptive gait with dual task).

 During adaptive gait and free gait with dual task, AD patients need more time to obtain environment information, plan the task, and process information, respectively. In both experimental condition, AD patients showed longer stride duration and reduced velocity. Moreover, patients increased stability during free gait with dual task (longer duration of the double support phase). For both experimental conditions, the AD patients used online adjustments [18] to perform the tasks. Locomotion in environments with obstacles, such as adaptive gait in this study, requires an adaptive ability to cross irregular terrain [19, 20], as well as a higher-attention demand [21]. Slow movement strategy, with a consequent increase in the time of vision for action planning, is used to increase time to explore and obtain relevant information [22]. Exteroceptive information is used to plan the action in advance [23]. Since AD patients have attention deficits [24, 25], walking more slowly was the conservative strategy to plan successfully the obstacle crossing action. Both tasks in free gait with dual task use the subcortical area during execution [25]. The divided attention for each task demands more of this area [3, 10, 11]. However, AD patients show executive task deficits [9, 26], impairing the execution of two concurrent tasks [1, 3, 4, 27]. Moreover, patients show impairment of speech-related areas [28]. Even so, the number of mistakes in the executive task during trials was high (37.6%), suggesting that both tasks depend on the same functional subsystem [25], leading to divided attention between tasks. Therefore, the slow gait velocity allowed more time for the cognitive system of the AD patients to try solving the problem of concurrent processing in the dual task during gait. Furthermore, the longer duration of double support is a strategy to compensate the low velocity, which, although allows greater control during movement, can cause gait instability [29–31]. 

 During adaptive gait with dual task, patients need anticipatory and online adjustments [18] to perform the task. In spite of higher number of adjustments in this task, their efficiency rate did not improve, increasing the mistakes in the secondary task (44.7%) and obstacle crossing (5.9%). Patients used the same strategy for the obstacle approaching and crossing phases, reducing stride velocity and duration and improving stability (higher basis of support and duration of double support). It is possible that obstacle crossing and performing a concurring executive task is too complex for this population, requiring more from its processing areas [8]. Furthermore, even while adopting a conservative strategy during task execution, the patients used a risky strategy when crossing with the support limb, reducing toe clearance and foot-obstacle distance after crossing (not significant). Again, the divided attention is a key factor for planning and online adjustments during dual task adaptive gait leading the subject to decrease toe clearance and increase the risk of falling.

 The experimental design of this study showed us a sequence in the difficulty degree of each task executed by AD patients. The similarity between free gait with dual task and adaptive gait with dual task suggests that dual task demands more from the cortical areas than obstacle crossing task for AD patients. Thus, due to prefrontal cortex deterioration there is a decrease in the executive ability, making it more difficult to divide the attention between two tasks [9, 26]. Therefore, we could suggest that free gait without dual task is the simplest, obviously, followed by adaptive gait without dual task, free gait with dual task, and adaptive gait with dual task. Furthermore, for our knowledge this is the first study that increases demands cognitive and motor during gait for AD patients. The motor changes happened due to high cognitive load, decreasing automated motor of gait [32], and increasing risk of falls [1, 25].

 Even with consistent and relevant results, this study has some limitations. The countdown secondary task demands, besides processing and planning by the frontal area, another motor component: counting out loud. The task, then, becomes even more demanding, with the patient performing two concurring motor tasks. Moreover, counting out loud can be a limiting factor on patient's velocity, which can drive the countdown according to the contact of foot with the ground. Furthermore, it would be interesting to have a group of healthy elderly people performing the same tasks to observe their behavior in relation to AD patients. Notwithstanding, our findings remain significant, since our comparisons had free gait without dual task as a baseline.

 In conclusion, the patients with AD walk more slowly during adaptive gait and free gait with dual task to have more time to obtain environment information and to plan the task and to process the information, respectively. Therefore, during adaptive gait with dual task, the patients used anticipatory and online adjustments to perform the task and need to improve the stability during the task, using more conservative strategies. Moreover, the increase of task complexity, such as adaptive gait with dual task, enhances cognitive load and risk of falls for AD patients.

## Figures and Tables

**Table 1:** General, clinical, and anthropometric characteristics of each patient.

	Sex	CDR	Age (years)	CDT (pts)	FAB (pts)	MMSE (pts)	Weight (kg)	Height (m)
A	F	1	66	6	9	18	84.0	1.61
B	M	1	88	8	18	24	77.1	1.64
C	M	1	73	4	17	26	71.2	1.57
D	F	1	81	4	15	17	63.7	1.52
E	F	1	83	8	12	20	75.3	1.60
F	F	1	88	4	11	18	45.5	1.53
G	F	1	77	8	16	22	58.7	1.46
H	F	1	69	5	15	19	54.2	1.50
I	F	2	76	9	10	17	68.0	1.56
J	M	1	81	7	14	24	67.1	1.66
K	F	2	77	4	9	13	54.2	1.54
L	M	1	81	4	9	13	56.1	1.55
M	M	1	82	4	12	18	67.8	1.68
N	F	1	78	9	17	23	47.1	1.49
O	F	1	83	6	9	18	57.1	1.40
P	F	1	77	3	14	11	83.1	1.63
Q	F	1	86	4	8	16	47.0	1.46
R	F	2	83	6	6	18	43.5	1.46
S	F	1	72	8	12	16	67.1	1.55

Mean ± SD			79.0 ± 6.1	5.8 ± 2.0	12.26 ± 3.5	18.4 ± 3.9	62.5 ± 12.4	1.55 ± 0.08

F: female; M: male; CDR: clinical dementia rating; CDT: clock drawing test; FAB: frontal assessment battery; MMSE: mini-mental state examination.

**Table 2:** Mean and standard deviation of the spatial-temporal parameters in the crossing stride.

	Adaptive gait without dual task	Adaptive gait with dual task	*P*-values
Single support duration (s)	1.32 ± 0.19	1.41 ± 0.25	0.004^d^
Double support duration (s)	0.47 ± 0.14	0.61 ± 0.24	0.001^d^
Stride duration (s)	1.79 ± 0.27	2.02 ± 0.44	0.001^d^
Stride velocity (cm/s)	118.53 ± 32.15	102.45 ± 37.71	0.001^d^
Horizontal distance of foot to obstacle-LL (cm)	78.5 ± 22.67	77.41 ± 24.91	0.746
Horizontal distance of obstacle to foot-LL (cm)	21.95 ± 15.91	21.21 ± 17.52	0.768
Toe clearance-LL (cm)	13.19 ± 4.64	12.82 ± 5.04	0.577
Horizontal distance of foot to obstacle-TL (cm)	40.14 ± 10.24	38.81 ± 9.92	0.235
Horizontal distance of obstacle to foot-TL (cm)	63.86 ± 14.64	59.09 ± 19.49	0.062
Toe clearance-TL (cm)	26.71 ± 9.2	23.97 ± 7.76	0.029^d^

LB: leading limb; TL: trailing limb. d: differences between adaptive gait without dualtask and adaptive gait with dual task.
